# Vignette of *Vigna* domestication: From archives to genomics

**DOI:** 10.3389/fgene.2022.960200

**Published:** 2022-10-21

**Authors:** Sachin Kumar Verma, Chandan Kumar Singh, Jyoti Taunk, Gayacharan, Dinesh Chandra Joshi, Sanjay Kalia, Nrisingha Dey, Amit Kumar Singh

**Affiliations:** ^1^ ICAR-National Bureau of Plant Genetic Resources, New Delhi, India; ^2^ Department of Biotechnology, University Centre for Research and Development, Chandigarh University, Mohali, Punjab, India; ^3^ ICAR-Vivekananda Institute of Hill Agriculture (Vivekananda Parvatiya Krishi Anusandhan Sansthan), Uttarakhand, Almora, India; ^4^ Department of Biotechnology, Ministry of Science and Technology, New Delhi, India; ^5^ Institute of Life Sciences, Bhubaneswar, Odisha, India

**Keywords:** Domestication, domestication-related traits, genomics, progenitors, *Vigna*, wild

## Abstract

The genus *Vigna* comprises fast-growing, diploid legumes, cultivated in tropical and subtropical parts of the world. It comprises more than 200 species among which *Vigna angularis*, *Vigna radiata*, *Vigna mungo*, *Vigna aconitifolia*, *Vigna umbellata*, *Vigna unguiculata*, and *Vigna vexillata* are of enormous agronomic importance. Human selection along with natural variability within these species encompasses a vital source for developing new varieties. The present review convokes the early domestication history of *Vigna* species based on archeological pieces of evidence and domestication-related traits (DRTs) together with genetics of domestication. Traces of early domestication of *Vigna* have been evidenced to spread across several temperate and tropical regions of Africa, Eastern Asia, and few parts of Europe. Several DRTs of *Vigna* species, such as pod shattering, pod and seed size, dormancy, seed coat, seed color, maturity, and pod dehiscence, can clearly differentiate wild species from their domesticates. With the advancement in next-generation high-throughput sequencing techniques, exploration of genetic variability using recently released reference genomes along with *de novo* sequencing of *Vigna* species have provided a framework to perform genome-wide association and functional studies to figure out different genes related to DRTs. In this review, genes and quantitative trait loci (QTLs) related to DRTs of different *Vigna* species have also been summarized. Information provided in this review will enhance the in-depth understanding of the selective pressures that causes crop domestication along with nature of evolutionary selection made in unexplored *Vigna* species. Furthermore, correlated archeological and domestication-related genetic evidence will facilitate *Vigna* species to be considered as suitable model plants.

## Introduction

The genus *Vigna* belongs to the family *Fabaceae*, which consists of more than 200 wild and cultivated species (Pratap et al., 2014). *Vigna* species are grown in tropical and subtropical regions of Asia, Africa, Australia, and the Pacific regions (Tomooka et al., 2002; Maxted et al., 2004). Currently, the genus *Vigna* is categorized into five subgenera, namely, *Ceratotropis*, *Plectrotropis*, *Vigna*, *Haydonia*, and *Lasiospron*, amongst which domesticated species are confined to only first three subgenera. *Vigna* species are economically important as they provide nutrition to humans and forage for animals in large parts of the tropical underdeveloped African and Asian countries. These include crops such as adzuki bean [*V. angularis* var. *angularis* (Wild) Ohwi and Ohashi], mung bean [*V. radiata* (L.) Wilczek], black gram [*V. mungo* (L.) Hepper], rice bean [*V. umbellata* (Thunb.) Ohwi and Ohashi], moth bean [*V. aconitifolia* (Jacq.) Marechal], cowpea [*V. unguiculata subsp. unguiculata* var. *spontanea* (Schweinf.)], and zombie pea [*V. vexillata* (L.) A. Rich] (Takahashi et al., 2016).

Mung bean and cowpea are the most important domesticated *Vigna* species in terms of global production. In the year 2020, cowpea had a production of 8.9 million tons (mt) covering 15 million ha of land worldwide (http://www.faostat.fao.org/faostat). Production of mung bean has increased at a rate of 2.5% annually in the recent decade. This increase can be bestowed to its short crop duration, which fits into different crop rotation cycles. Therefore, production of mung bean has reached 5.3 mt on about 7.3 million ha coverage during the year 2020 (Nair and Schreinemachers, 2020). Despite their immense agricultural value, the concentrated crop improvement efforts in *Vigna* species are lagging behind as compared to other legumes. This may be attributed to poorly characterized genetic mechanisms governing the domestication of *Vigna* species. Furthermore, *V. vexillata*, which is closely related to cowpea, is an underutilized crop originated in Africa. It shows high resistance against insects and pests of cowpea and can therefore, be used in breeding programs for disease and insect resistance in cowpea (Ogundiwin et al., 2005).

Plant domestication is a complex evolutionary phenomenon where humans select traits and species that lead to desired morpho-physiological changes in plants under various environmental conditions. The trait selection process for domestication—which is usually similar across many plant species—influences evolution such that plants can be distinguished into domesticated taxa and their wild types. The phenotypic and genotypic changes brought about in plants during the process of domestication are known as the “domestication syndrome” (Hammer, 1984). Plants such as wild *Vigna* species that have survived several harsh environmental conditions have witnessed induction of adaptive mechanisms. This natural survival process also enhances the domestication syndrome and consequently affects morpho-physiological and molecular changes in plants. Thus, it has been observed over the years that knowledge of the nature of multiplicity of processes related to the domestication syndrome and natural evolution is vital in understanding the origins of domestication (Abbo et al., 2014).

Identification of the origin of domestication of plant species including *Vigna* requires archeological evidence of evolution and domestication, which is supported by morpho-physiological traits related to domestication together with genetic variation identified using molecular markers. Some of the important domestication-related traits (DRTs), such as loss of seed dispersal, plant architecture, increase in pod and seed size, early flowering, and reduction of pod shattering, have been used in recent years to identify the variability and adaptation from its wild *Vigna* species (Xu et al., 2000; Isemura et al., 2012; Lo et al., 2018). Furthermore, gathering in-depth knowledge of the desired gene(s)/genomic regions and molecular markers that govern these DRTs is an essential step in breeding and crop development programs. Molecular markers play an important role in genetic diversity analyses that provide information about genomic variation and genetic relationships among the genotypes (Tan et al., 2022). Genus *Vigna* was primarily characterized based on morpho-physiological traits; however, these traits are influenced by diverse environmental conditions (Meglic and Staub, 1996). Therefore, molecular markers which are robust, reliable, and, importantly, not impacted by environmental conditions are preferable for genetic characterization and for studying the process of domestication in crop plants. They are also used in linkage map construction, in establishing archeological evidence, construction of population structure, and to figure out the origin of genetic diversity in various crops (Kaga et al., 2008; Andargie et al., 2011, 2014; Yundaeng et al., 2019). Molecular markers have also been used for studying molecular phylogenetic relationship of *Vigna* with *Phaseolus* and *Glycine* species using restriction fragment length polymorphism (RFLP) (Fatokun et al., 1993), simple sequence repeats (SSRs) (Tangphatsornruang et al., 2009), and internal transcribed spacer (ITS) sequences (Goel et al., 2002). A recent approach of genotyping using a single nucleotide polymorphism (SNP) marker has emerged as a powerful technique, as these markers are abundant and cover the whole plant genome (Varshney et al., 2007; Deulvot et al., 2010). Furthermore, advancement in next generation sequencing (NGS) technologies has also helped in development of functional molecular markers *via* exploring the whole genomes. In *Vigna*, it has helped in generation of different markers, such as SNPs and SSRs, which have been used in molecular mapping and marker-assisted breeding. Higher cross transferability rates of these markers in *Phaseolus vulgaris* and *Glycine max* have also allowed understanding of chromosomal synteny (Tangphatsornruang et al., 2009).

*Vigna* species are suitable model plants for genetic studies due to their relatively small genome size ranging from 416 to 1,394 Mbp, together with fast growth rate, adequate seed production, short life span, high intraspecific variation for stresses, and retained cross-compatibility. *Vigna* also have the potential to fulfill the global pulse demand, yet it is unfortunate that these species are amongst the unexplored and underutilized crops till date. Furthermore, existing scenarios of climate change such as increase in temperatures, seasonal shift, flooding, and drought have largely affected the production of *Vigna* crops (Varshney et al., 2015). In this review, the significance of domestication syndrome, mechanisms, and how different factors influence this important trait contributing toward the domestication of the cultivated *Vigna* species are discussed. The recent developments in morpho-physiological, genetics, and genomics that have contributed toward understanding the complex mechanism of domestication in *Vigna* species are also presented. The knowledge gained on this very important aspect of *Vigna* species is essential for designing suitable breeding strategies in the existing scenario of climate change and natural vagaries, which affect yield and productivity of *Vigna* species globally.

### Domestication-related traits in *Vigna*

During the domestication process, human selection causes genetic alteration in morphological and agronomical traits. Thus, differences in phenotypic traits of wild and cultivated varieties are observed, which are defined as the ‘domestication syndrome’ (Abbo et al., 2014). In case of *Vigna*, most of these traits are associated with pod shattering, seed dormancy, days to flowering, pod length, seeds/pod, leaf length, leaf width, seed weight, etc. Knowledge about the genetics and physiological aspects of these traits will allow the breeders to use them in identification of associated genes. Pod shattering is a major feature of wild to disperse seeds over long distances. However, this trait causes severe yield loss during harvesting (Maity et al., 2021). Pod shattering has been used as an important DRT for depicting QTLs in several studies on *Vigna* (Isemura et al., 2007; Andargie et al., 2011; Isemura et al., 2012; Andargie et al., 2014; Yundaeng et al., 2019). Seed dormancy is another common DRT. Naturally, it is crucial that the seeds germinate at the right time frame for better survival, seed dispersal, and environmental compatibility. However, extended seed dormancy in crop plants is deleterious for the overall yield, as it causes uneven germination (Soltani et al., 2021). Seed dormancy is categorized into two categories: physical seed dormancy, caused by improper imbibition of water, and physiological seed dormancy, caused due to imbalance in plant hormones such as gibberellic acid (GA) and abscisic acid (ABA) (Finch-Savage and Leubner-Metzger, 2006). Several studies have deduced QTLs and candidate genes for seed dormancy in *Vigna* species (Isemura et al., 2012; Laosatit et al., 2012).

The flowering time in legumes, which is influenced by several environmental conditions, is an important factor for plants’ adaptation to different eco-geographical locations. It is governed by several genetic mechanisms, influencing photoperiod and vernalization response (Weller and Ortega, 2015). Variation in flowering time of wild and cultivars—where cultivars having a shorter time to flower than wild accession—has been considered a crucial trait for domestication and crop improvement. Studies on flowering time as a domestication trait in *Vigna* to detect associated QTLs\candidate genes have been conducted by Isemura et al. (2012), Andargie et al. (2014), Lo et al. (2018), and Yundaeng et al. (2019). Apart from these DRTs in *Vigna*, several other traits that are related to plant morphology, which can visually distinct the wild from its cultivars, such as pod length, seeds/pod, leaf length, leaf width, and seed weight, have been used in several studies to identify associated QTLs (Isemura et al., 2007; Kaga et al., 2008; Andargie et al., 2011; Andargie et al., 2014; Li et al., 2017; Lo et al., 2018). The following section of review describes the different aspects of domestication in different *Vigna* species in detail.

### *Vigna unguiculata* (L.) Walp

Cowpea (*V. unguiculata* [L.] Walp.), a diploid (2*n* = 22) legume belonging to the *Fabaceae* family, has an estimated genome size of 640.6 Mbp (Lonardi et al., 2019). It is amongst the abiotic stress resilient crops grown worldwide that provide food and nutritional security, especially to the people of developing countries such as sub-Sharan Africa. It is a rich source of protein to humans and also provides fodder for animals. Its cultivation has spread across the regions of Asia, Europe, United States, and Central and South America (Lonardi et al., 2019).

#### Origin and taxonomy

The taxonomic status of cultivated cowpea was disputed for a long time; however, years of research have led to the categorization of cultivated cowpea as *V. unguiculata ssp unguiculata.* It is considered as a species complex with varying number of sub-species. Currently, 11 wild sub-species are recognized within the *V. unguiculata* species complex including spp. *aduensis*, ssp. *alba*, ssp. *baoulensis*, ssp. *burundiensis*, spp. *letouzeyi*, spp. *pawekiae*, spp. *kindtiana*, ssp. *protracta*, ssp. *pubescens*, ssp. *stenophylla*, and ssp. *tenuis* (Pasquet, 1993; Pasquet, 1993; Padulosi and Ng, 1997). Among these, *V. unguiculata* ssp. *dekindtiana*, var. *spontanea* is considered as the immediate progenitor of cultivated cowpea (Pasquet and Padulosi, 2012). It is essentially a weed, which is easily crossable with cultivated cowpea and abundantly found on fringes of the cultivated lands all over Africa (Coulibaly et al., 2002; Feleke et al., 2006). Gene pool studies in *V. unguiculata* using classical breeding methods have suggested that its primary gene pool consists of cultivated and autogamous spontaneous forms and wild allo-autogamous forms, whereas the secondary gene pool contains only the allogamous forms (Pasquet and Baudoin, 1997). This conclusion about the *V. unguiculata* gene pool was further supported by Kouadio et al. (2007).

#### Domestication and global spread

**FIGURE 1:**
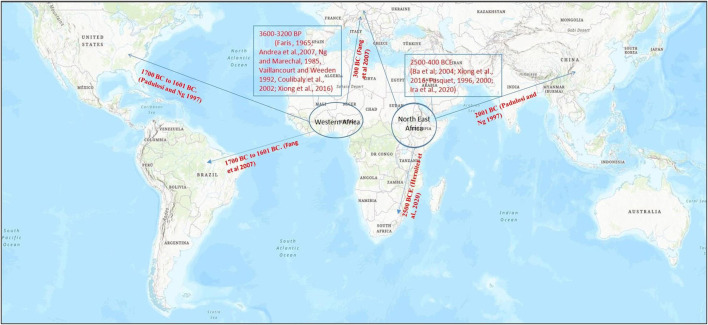
Map representing cowpea (*Vigna unguiculata*) origin of domestication (encircled) and its spread (arrow) to different parts of the world based on archeological evidences and genetic studies for domestication-related traits.

The cowpea’s domestication history has been thoroughly researched among all the cultivated *Vigna* species; however, there is no consensus on its exact domestication location. Vavilov (1926) was the first to propose Ethiopia as the major center of its domestication, with India and China as the minor centers. Subsequent research works revealed that cowpea’s wild relatives were only found in Africa, ruling out India and China as the possible domestication sites (Figure 1). Many ensuing studies have added to our understanding of cowpea domestication (Faris, 1965; Herniter et al., 2019); where Faris (1965) was among the first ones to present comprehensive view of cowpea domestication based on a review of previous studies and his own extensive work on the morphological descriptors. He proposed that cowpea domestication might have occurred either in West or Central Africa. Later, it was concluded that domestication of cowpea took place in one of the two regions: West Africa or Northeastern Africa (Ba et al., 2004; Xiong et al., 2018). The theory that favored cowpea domestication in the West African region was supported by evidences such as huge morphological variability among the cultivated cowpea varieties, existence of wild hybrids from crosses between wild and cultivated forms, and the presence of cowpea seeds in the archaeological evidences (Ba et al., 2004; D’Andrea et al., 2007). Moreover, the theory also gained support from a molecular study that showed similarities in the chloroplast DNA of the cultivated and wild cowpea varieties of Nigeria. However, this theory was later rejected as the isoenzymatic and ethnobotanical studies did not reveal diversity in wild cowpea forms of West Africa (Vaillancourt et al., 1993; Paquet, 1996; Feleke et al., 2006).

On the other hand, theory of cowpea domestication in the Northeastern African region relied on two main points: 1) absence of any real ecotype of the wild cowpea in the West African region and 2) huge variability in wild cowpea forms in the region ranging from Ethiopia to Southern Africa (Baudoin and Maréchal, 1985). Furthermore, ethnobotanical and linguistic group studies have also supported Northeastern African theory of domestication, where cowpea was found to be related with two linguistic groups, namely, Chadic and Nilo-Saharan language speaking populations that are common to Northeastern Africa (Pasquet and Fotso 1994). This hypothesis also found support from the genetic characterization studies of worldwide collection of cowpea landraces and wild genotypes using molecular markers (Pasquet and Fotso, 1994; Pasquet, 2000; Coulibaly et al., 2002).

In addition to detailed understanding of origin and likely site of cowpea domestication, tracking its spread across the world is critical for developing strategies for its genetic improvement. It is believed that spread of cowpea in America took place between the 16th and the 17th centuries, whereas in Asia, it was around the third millennium BC (Padulosi and Ng 1997). Fang et al. (2007) highlighted the introduction of cowpea in Europe in 300 BC. Furthermore, they suggested that cowpea would have exported to new world by Portuguese and Spanish in the 17th century. Later, cowpea was introduced in the Southern United States. This information also exhibits the overall spread of cowpea in the colder latitudinal region from 35° N to 30° S. However, it was also spread over the hot areas of Asia and Oceania (Watt et al., 1985). Recently, Herniter et al. (2020) reconstructed the spread of cowpea around the globe using genetic, textual, and taxonomic evidences. They concluded that cowpea was most likely domesticated in Africa approximately 2500 BCE, and by 400 BCE, it had spread to all of the old world’s contemporary growing zones, including sub-Saharan Africa, Mediterranean basin, India, and Southeast Asia.

#### Genetic diversity in global cowpea collections

Understanding the extent and pattern of genetic diversity in the global cowpea landrace collections and its ancestors can aid in tracking its domestication history as well as migration patterns in different parts of the world. Presently, few studies have explored genetic diversity in cowpea using large collections of landraces from different regions of the world by utilizing SNP markers (Huynh et al., 2013; Xiong et al., 2018; Herniter et al., 2020). Huynh et al. (2013) deployed SNPs to analyze genetic diversity in a global cowpea landrace collection of 422 genotypes along with 46 genotypes from its ancestral wild species from Africa. They observed two gene pools in the African landrace collection, which represented West African and East African regions, respectively. Each of the African landrace gene pool was closely related to wild cowpea that corresponded to the same geographical region. This suggested that divergent domestication processes might have resulted in the formation of two gene pools. Furthermore, they also reported relatively wider genetic diversity in the East African gene pool than that in the West African gene pool. Based on this observation, it is suggested that there would have been a single domestication event of cowpea in the East African region, from where it may have reached West Africa *via* human migration and have undergone directional selection to give another gene pool with relatively narrow diversity. In another study, 768 cowpea accessions from 56 countries representing various regions of the world were genotyped using the genotyping-by-sequencing (GBS) approach. A total of 1,048 SNPs were discovered, which revealed three well-distinguished gene pools in this set. Among all the regions, rich diversity was observed in the African continent, with the East African region having highest diversity, whereas South and West populations had slightly lower diversity. These findings were consistent with previous research work, which suggested that the East African region is the center of domestication, and subsequent human migration may have resulted in another cowpea gene pool in Western Africa (Xiong et al., 2018). Recently, Muñoz-Amatriaín et al. (2021) studied molecular diversity in a mini core set of 368 cultivated cowpea accessions, representing worldwide diversity using 51,128 SNPs. Genetic assignment and principal component analyses using SNP data grouped mini core set accessions into six sub-populations, mainly differentiated according to the geographic origin and cultivar groups. Each group has accessions from West African regions, which was expected, as previous studies have shown highest diversity within this region (Muñoz-Amatriaín et al., 2021). Thus, center of diversity for cowpea is in the West African region may not be necessarily the center of its domestication.

#### Genetics and genomics of domestication

The advent of advanced genomic technologies has made genotyping data generation easier and cheaper, accelerating gene mapping studies in crops including cowpea. Mostly, gene mapping studies in cowpea have mainly focused agronomic traits; however, in the past decade few studies have mapped DRTs in this legume (Andargie et al., 2011; Kongjaimun et al., 2012b; Andargie et al., 2014; Lo et al., 2018) (Table 1) and its related ssp. *sesquipedalis* (Kongjaimun et al., 2012a; Suanum et al., 2016). Andargie et al. (2011) reported six quantitative trait loci (QTLs) for seed size and four QTLs related to pod shattering in a F_7_ generation recombinant inbred lines (RILs) population of 159 individuals developed from a cross between 524B, a California-based black-eyed type and a unique wild perennial cowpea *V. unguiculata* subsp. *unguiculata* var. *spontanea* from coastal Kenya. For both the traits, QTLs were clustered in two regions on linkage group (LG) 1 and LG 10, which made their distinction easier for transferring desirable traits from the wild species. In another study, Andargie et al. (2014) dissected QTLs for 10 different DRTs using the same mapping population (159 F_7_ RILs). A total of 17 QTLs were identified for various DRTs, such as reduced seed dormancy and increased seed germination (1 QTL each; LG 1), seed coat permeability (2 QTLs; LGs 2 and 10), seed size (7 QTLs; LGs 1, 2, 3, 7, and 10), pod shattering (1 QTL; LG 5), pod fiber layer thickness (5 QTLs; LGs 1, 5, 6, and 10), days to flowering (3 QTLs; LGs 1, 2, and 7), pod color (1 QTL; LG 2), and ovule number (2 QTLs; LGs 1 and 3). Moreover, it was reported that QTLs for two or more DRTs were co-localized, implying their non-random distribution (Andargie et al., 2014). The finding concluded that cultivated cowpea retained several DRTs QTLs with a small effect of phenotypic variance explained (PVE) < 10% that differentiated wild from the cultivated ones. Another study by Lo et al. (2018) mapped QTLs for DRTs in a biparental mapping population obtained from a cross of IT99K-573-1-1 (cultivated) and TVNu-1158 (wild) genotypes, where a total of 16 QTLs were identified for nine DRTs, which were distributed on 7 out of 11 cowpea chromosomes. Furthermore, putative candidate genes were identified for two major pod shattering QTLs, that is, *CPshat3* and *CPshat5*. Two candidate genes were located in the *CPshat3* QTL region, namely, *Vigun03g306000*, which encodes for a NAC domain transcription factor (NAC007) and *Vigun03g302600* encoding C2H2 Zinc family protein. These were considered as the probable candidates governing pod shattering in the wild cowpea (Lo et al., 2018). The identified NAC transcription factors were involved in secondary cell wall synthesis, thereby affecting pod shattering resistance in wild cowpea. This conclusion was consistent with a previous report in soybean, which also found involvement of a NAC transcription factor family gene in regulating pod shattering resistance *via* activating secondary cell wall synthesis (Liljegren et al., 2000). Additionally, a genome-wide association study (GWAS) approach has also enabled mapping of DRTs in cowpea using diverse set of genotypes (Muñoz-Amatriaín et al., 2021). In this study, the GWAS analysis using a mini core set of cowpea germplasm identified many loci for flowering under short as well as long days. Furthermore, the genomic regions coincided with those identified in previous studies based on biparental population. Watcharatpong et al. (2020) performed fine-mapping of DRT QTL regions which were previously found by Suanum et al. (2016). These QTLs were associated with pod fiber content, that is, cellulose (*qCel7.1*), hemicellulose (*qHem7.1*), and lignin (*qLig7.1*), identified on LG 7 using F_2_ and F_2:3_ mapping populations derived from a cross between JP81610 (cultivated yardlong bean) and TVnu-457 (wild cowpea). This study resulted in identification of one major and one minor QTL that governed pod fiber contents. Also, the same region was found to be associated with QTL confined to pod shattering. Amongst several genes associated with this region, two genes encoding beta glucosidase [*Vigun05g266600* (*VuBGLU12*)] and transcription factor MYB26 [*Vigun05g273500* (*VuMYB26b*)] were identified. These genes are also related to dormancy and pod shattering in different *Vigna* species such as moth bean (Yundaeng et al., 2019).

**TABLE 1:** Common major and minor DRT-associated QTLs detected in *Vigna* species.

	Domestication-related traits (DRTs)													
Species name	Pod dehiscence (PDT)	Seed dormancy (SDWA/SDP)/seed coat permeability (%)	100 seed weight (100SW)	Stem length 10 node (STL10/ST10I)	Days-to-flowering (FLD)	Pod maturity (PDDM)	Seed length (SDL)	Seed width (SDW)	Seed thickness (SDT)	4th internode (ST4I)	7th internode (ST7I)	Stem length (STL/STLW)	Stem thickness (STT)	References
Moth bean (MO)	LG1 (87), LG7 (7)	LG1 (134), LG9 (42)	LG1 (15), LG2 (27)LG3 (56), LG4 (30), LG7 (37),LG8 (68), LG10 (33)	LG2 (81), LG7 (6)	LG2 (67), LG6 (71)	LG2 (81), LG4 (5)	LG1 (25), LG2 (28), LG4 (26), LG7 (14), LG8 (70), LG10 (34)	LG4 (28), LG7 (31), LG10 (6)	—	—	—	—	LG3 (41)	Yundaeng et al. (2019)
Azuki bean (AZ)	LG7 (11.3)	LG1 (36.1), LG4 (11.8), LG9 (2.6)	LG1 (28.5), LG2 (45.5), LG5 (2.3), LG9 (6.8)	LG4 (52.7), LG7 (24.4), LG9 (8.5)	LG4 (57.6)	—	LG1 (20.6), LG2 (43.9), LG3 (38.2), LG7 (21.7), LG8 (43.4), LG9 (10.3), LG11 (20.6)	LG1 (93.1), LG2 (44.7), LG3 (44.4), LG9 (8.3)	LG1 (90.3), LG2 (47.9), LG5 (3), LG9 (8.8)	LG1 (90.4), LG2 (45.9)	LG1 (83.1), LG2 (26.5), LG3 (11), LG4 (64.5), LG5 (14.6), LG7 (13.8), LG9 (14.7)	LG1 (88), LG2 (25.9), LG4 (63.8), LG5 (14.2), LG7 (19.2), LG9 (6)	LG4 (52.2), LG6 (0.9), LG9 (22.1)	Isemura et al. (2007)
Rice bean (RB)	LG7 (14.52)	LG2 (68.11), LG3 (33.36), LG4 (82.52), LG8 (61.37), LG10 (24.28)	LG1 (83.96), LG2 (54.10), LG3 (23.35), LG4 (64.65), LG5 (7.66), LG7 (34.94)	LG1 (95.93), LG7 (0.00)	—	LG4 (78.19)	LG1 (78.50), LG2 (52.28), LG4 (63.86), LG5 (35.41), LG7 (29.32)	LG1 (70.58), LG2 (55.61), LG3 (23.35), LG4 (63.60), LG5 (1.37), LG7 (36.71), LG11 (27.42)	LG1 (83.96), LG2 (53.32), LG3 (23.35), LG4 (63.72), LG5 (6.26), LG7 (41.69)	LG1 (85.99), LG7 (14.61)	LG1 (99.10), LG7 (10.62)	LG1 (93.50), LG5 (24.29), LG7 (11.13)	LG9 (47.45)	Isemura et al. (2010)
Mung bean (MB)	LG1 (48.2), LG7 (8.7)	LG1 (60.0), LG2 (55.8), LG3 (19.0), LG4 (21.0)	LG1 (64.6), LG2 (58.8), LG3 (29.2), LG7 (1.5), LG8 (46.6), LG11 (22.6)	LG2 (63.1), LG6 (38.1), LG9 (17.6), LG10 (40.8)	LG2 (62.1), LG4 (75.7), LG6 (30.1), LG11 (15.1)	LG2 (53.9), LG4 (74.6), LG6 (30.8), LG7 (21.8), LG9 (17.8), LG11 (17.1)	LG1 (68.3), LG2 (58.2), LG3 (32.1), LG7 (5.8), LG8 (46.6), LG11 (29.4)	LG1 (28.8), LG2 (57.0), LG3 (27.1), LG7 (4.9), LG8 (47.2), LG11 (26.6)	LG2 (62), LG3 (27), LG7 (4), LG8 (47.5), LG11 (33.2)	—	LG10 (45.2)	LG1 (3.1),LG2 (68.8),LG3 (13.8), LG6 (42.1), LG9 (14), LG10 (46)	LG2 (71.1)	Isemura et al. (2012)
Cowpea (CO)	LG1 (93.4), LG4 (24.5), LG7 (9.3), LG9 (58.1)	LG1 (90.2), LG2 (2.2), LG4 (46.9), LG7 (30.8), LG8 (82.8), LG11 (17.5)	LG1 (89.0), LG2 (2.9), LG3 (11.5), LG4 (24.3), LG5 (64.9), LG6 (38.7), LG7 (22.9), LG8 (23.7), LG10 (28.9), LG11 (31.9)	LG1 (104.6), LG2 (56.9), LG3 (32.1), LG4 (81.6), LG5 (67.0), LG6 (94.9), LG7 (36.9), LG8 (68.2), LG9 (64.3), LG10 (59.5), LG11 (5.7)	LG1 (17), LG2 (65.5), LG4 (81.6), LG5 (50.3), LG6 (36.6), LG7 (54.1), LG8 (36.1), LG9 (50.8), LG10 (39.2), LG11 (40.4)	LG1 (28.3), LG2 (78.3), LG3 (2), LG4 (52.9), LG6 (57.7), LG7 (59.7)	LG1 (94.9), LG2 (56.5), LG3 (8.6), LG4 (35), LG5 (15.9), LG6 (34.5), LG7 (46.3), LG8 (56.2), LG11 (34.4)	LG1 (93.2), LG2 (15.6), LG3 (16.8), LG4 (58.3), LG6 (61.2), LG7 (21.6), LG8 (29.9), LG10 (23.3)	LG1 (20.3), LG2 (70.7), LG3 (16.4), LG4 (3.8), LG6 (39.4), LG7 (43), LG8 (20.4), LG9 (51.4), LG10 (38.6), LG11 (29.2)	—	—	LG1 (62.9), LG2 (51.0), LG3 (3.3), LG5 (35.7), LG6 (61.2), LG8 (39.0), G10 (59.9)	LG1 (87.5), LG2 (86.9), LG3 (83.2), LG4 (30.8), LG6 (56.0), LG7 (42.0), LG9 (19.5), LG10 (63.2), LG11 (35.5)	Kongjaimun et al. (2012b)
Common QTLs	LG1 (MO, CO), LG7 (MO, AZ, RI, MU, CO)	LG3 (RI,MU), LG4 (AZ,MU)	LG1 (RI, CO and MO, AZ), LG2 (AZ, RI, MU), LG3 (RI, MU and RI, CO), LG4 (MO, CO), LG10 (MO, CO), LG11 (MU,CO)	LG1 (RI, CO), LG7 (MO, RI and AZ, CO), LG9 (AZ,MU)	LG2 (MO, MU), LG4 (MU,CO), LG6 (MU,CO)	LG4 (RI,MU)	LG1 (RI, MU and AZ, CO), LG2 (RI, MU, CO), LG3 (AZ,MU), LG7 (AZ, RI, CO), LG7 (MO, AZ and MO, MU), LG8 (AZ, MU and MU, CO), LG11 (AZ, MU, CO)	LG1 (AZ, CO), LG2 (AZ, RI, MU), LG3 (RI, MU, CO),LG4 (CO, RI), LG7 (MO, RI), LG11 (RI, MU)	LG1 (AZ, RI), LG5 (AZ, RI), LG3 (RI, MO, CO)	LG1 (AZ, RI)	LG1 (AZ, RI), LG7 (AZ, RI)	LG1 (AZ, RI), LG5 (AZ, RI), LG7 (AZ, RI), LG9 (AZ, MU), LG10 (MU, CO)	LG9 (AZ, CO)	

LG, linkage group; QTL, quantitative trait loci; moth bean, MO; azuki bean, AZ; rice bean, RB; mung bean, MB; CO, cowpea.

### *Vigna angularis* var. *angularis* (Wild) Ohwi and Ohashi

Azuki bean (*V. angularis* var. *angularis*) is a diploid crop (2*n* = 2× = 22), rich in protein, phenolics, flavonoids, vitamin A, vitamin B, iron, zinc, and folate and is also acknowledged for its low fat content (Amarowicz and Pegg, 2008; Yao et al., 2012). Presently, azuki bean is considered as a conventional legume crop, which is grown in 30 countries of Eastern and Northern Asia, where Japan and China are among its largest producer around the world (Cheng and Tian, 2008).

#### Origin and taxonomy

**FIGURE 2:**
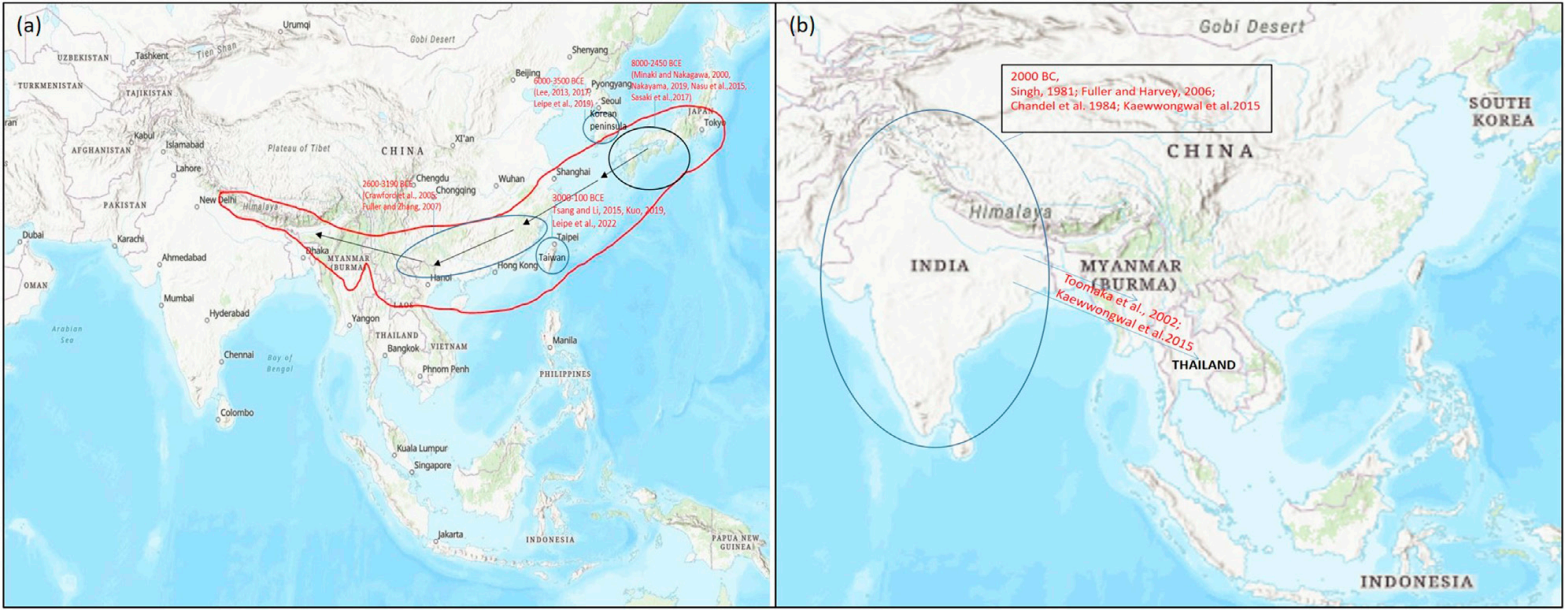
Map representing the origin of domestication of **(A)** azuki bean (*Vigna angularis*) and **(B)** black gram (*Vigna mungo*) (encircled) and their spread (arrow) to different parts of the world based on archeological evidences and genetic studies for domestication-related traits. Map source: https://www.arcgis.com/index.html.

Yabutsuru-azuki [*V. angularis* (Willd.) *nipponensis* (Ohwi) Ohwi *et* Ohashi] is considered as the probable progenitor of azuki bean which is widely grown in East Asia and is spread across Japan, China, and upto the fringe of the Himalayas (Tomooka et al., 2002). This region is also considered as the center of its domestication (Smartt, 1976). Several phylogenetic studies have identified ambiguity in its specific origin within East Asia (Yang and Han, 1994; Zong et al., 2003; Zong et al., 2003; Kaga et al., 2008). However, on the basis of recent archaeological findings, domestication of azuki bean (*V. angularis* var. *angularis*) is staged in East Asia (Lee et al., 2007) (Figure 2).

#### Domestication and global spread

Archaeological collections from preliminary studies have showed long-term evolution (8,000–100 BP) of azuki bean. In 1992, *V. angularis* var. *nipponensis* was presumed as the wild ancestor of adzuki bean, which was generally distributed in Japan, Korean peninsula, China, Taiwan, Nepal and Bhutan (Yamaguchi, 1992; Vaughan et al., 2004). Various studies have supported that the domestication of azuki bean has occurred in various regions of Korea (Lee, 2013; Lee, 2017; Leipe et al., 2021), China (Crawford et al., 2005; Fuller and Zhang, 2007), and Japan (Minaki et al., 2000; Nakayama, 2019; Nasu et al., 2015) (Figure 2A).

Morphological changes in seed shapes have revealed that cultural changes of azuki bean begun as early as 5,300–4,800 cal. BP. (Lee et al., 2007). In earlier studies it was reported that cultivated, wild and weedy adzuki bean existed as a complex crop as evidenced by the seeds found in the archeological site of Japan dated 4,000 years ago (Maeda, 1987; Vaughan et al., 2004; Yano et al., 2004). In 2006, Crawford predicted that adzuki bean is a crop of China and Korea. Furthermore, Liu et al. (2013) proposed that the adzuki bean was domesticated in China about 1,200 years ago. In a recent report by Leipe et al. (2021), it was suggested that apart from different evidences suggesting domestication of azuki bean in Japan, Korea and China dated ca. 4,000 and 1000 BCE; prehistorically Taiwan may have been its independent center of cultivation/domestication. This observation is supported by different archaeobotanical records recovered from Sanbaopi, an archaeological site of Taiwan (Leipe et al., 2021).

#### Genetic diversity in azuki bean collections

For understanding the adzuki bean genetic diversity, 146 *V. angularis* var. *angularis* and var. *nipponensis* germplasm collected from six Asian countries were dissected using amplified fragment length polymorphism (AFLP) (Xu-xiao et al., 2003). The study revealed seven distinct evolutionary groups on the basis of origin which were named accordingly. The study also revealed that there are several centers of origin of cultivated adzuki beans. Initially they might have been domesticated involving four progenitors at nearly three geographical origins, that is, China, Japan and West Himalayan range (Xu-xiao et al., 2003).

Molecular markers such as AFLP (Xu et al., 2000) and random amplified polymorphic DNA (RAPD) (Mimura et al., 2000) were used to genetically characterize wild, weedy and cultivated genotypes of azuki bean. Genotyping through AFLP marker showed that wild and weedy relatives have greater genetic variation than cultivars. Furthermore, weedy azuki beans, have a distinct ecotype, however they showed more relatedness with cultivars than the wild types. Therefore, they can be efficiently utilized in breeding programs for crop improvement (Xu et al., 2000). Similar findings were reported by Kang et al. (2015) while accessing 50 accessions of azuki bean which comprised of wild, semi-wild, landraces and cultivars. Semi-wilds were found closer to cultivars than the wild accessions, denoting their involvement in domestication as a preliminary landrace.

Doi et al. (2002) used intergenic region of chloroplast DNA and ribosomal DNA ITS, whereas Tomooka et al. (2002) utilized AFLP for molecular evaluation, which exhibited that cultivated azuki bean is related to *V. tenuicaulis* found in Thailand and Myanmar along with *V. nepalesis* found in the foot hills of Himalayas. On the other hand, Zong et al. (2003) used AFLP markers to deduce genetic diversity within cultivated and wild accessions from Bhutan, China, India, Japan, Korea, and Nepal. The result inferred that azuki bean domestication occurred twice, that is, in the Himalayan region of Southern Asia and in Northeast Asia. Xu et al. (2008) used 13 SSRs to dissect genetic diversity of 616 azuki bean accessions comprising of cultivated and wild accessions from eight Asian countries. Cultivated azuki bean from China, Japan and Korea depicted highest gene diversity level indicating East Asia as the center of its gene diversity.

#### Genetics and genomics of domestication

Genetics of DRTs in case of azuki bean was studied initially by Han et al. (2005). They developed mapping populations by crossing *V. nepalensis* (JP107881-Nepal) with *V. angularis* (JP81481-Japan). The BC_1_F_1_, F_2_, and F_2:3_ populations were used for the construction of linkage map using 205 SSR, 94 RFLP, and 187 AFLP markers, where total marker span was 832.1 cM. This linkage map was further used by Isemura et al. (2007) to detect several QTLs associated with 33 different DRTs. Most of the DRTs were mapped on LGs 1, 2, 4, 7 and 9. QTLs related to seed size, lower stem length, pod size and, germination efficacy were found to be present on LGs 1 and 2, while that for seed size, upper-stem length, pod size and maximum leaf size were found on LGs 7 and 9. In another study conducted by Kaga et al. (2008), a linkage map was developed using F_2_ mapping population by crossing wild azuki bean accession (JP110658) with a cultivar (JP109685 cv. Kyoto-Dainagon). A total of 316 adzuki bean SSRs, 170 common bean SSRs and 45 cowpea SSRs were used to screen for polymorphisms. In total 162 QTLs related to 46 DRTs were mapped of which, chromosomes seven and nine consisted of most of the traits (Kaga et al., 2008).

Draft genome investigation of 50 azuki bean accessions including 11 genotypes each of wild, semi-wild, and improved varieties along with 17 landraces revealed that the semi-wild adzuki bean is closer to the cultivated adzuki bean than wild adzuki bean (Kang et al., 2015). Similar observation was also made by Yang et al. (2015). Hence, it is considered that semi-wild adzuki bean might have played a significant role in adzuki bean domestication. In the same year, Kang et al. (2015) published a draft genome of adzuki bean, where they used 133 F_4_ lines derived from a cross between *V. angularis var. angularis* (Gyeongwon) and the wild species *V. nakashimae* which covered 75% of the estimated genome. In total, 4524 SNP sites were found among which 814 SNPs were used for the construction of 11 LGs after filtration (Kang et al., 2015). In another study, 26 agronomic QTLs and five qualitative trait genes related to pigmentation were mapped on linkage map using 1,571 SNPs. These SNPs were obtained from restriction site–associated DNA (RAD) sequencing of 150 F_2_ population derived from adzuki bean cultivar (Ass001) and a wild adzuki bean (accession CWA108) (Li et al., 2017). Recently, fine-mapping of pod shattering factor using SSRs and INDELs was performed by Takahashi et al. (2020) using backcross populations derived from a cross between JP81481 (cultivated azuki bean) and JP107881 (wild relative). The linkage map obtained for azuki bean and yardlong bean was narrowed down to 4 kbp and 13 kbp, respectively. The candidate genomic region was found to be associated with an important DRT, that is, pod shattering. It contained *MYB26* gene that promotes secondary wall thickening and development of pod sclerenchyma which enhances pod shattering. Therefore, lower expression or loss of function of this gene can increase the shattering resistance. Furthermore, the study also suggested that independent domestication process in both the legumes (azuki bean and yardlong bean) has selected similar locus associated with pod shattering. These studies revealed that majority of the QTLs and genes related to DRTs in case of adzuki bean are present on LG/chromosomes 1, 2, 3, 4, 6, 7, 8, 9, 10, and 11 (Table 1). Furthermore, clustering analysis showed that the protein set of adzuki bean is closely related to *Arabidopsis thaliana*, *Medicago truncatula*, *Oryza sativa*, and *G. max.* Candidate genes *VaAGL*, *VaPhyE*, *VaAP2*, *VaAP2/ERF.81*, *VaAP2/ERF.82*, and *VaUGT* related to traits such as pod maturity, flowering time, 100 seed weight, and black seed coat were identified on different chromosomes. The identified QTLs and associated markers can be used in marker-assisted selection and breeding for DRTs in adzuki bean.

### *Vigna mungo* L. Hepper

Black gram (*V. mungo* L. Hepper) is a self-pollinating, annual diploid crop (2n = 2× = 22) which is commonly known as urad, urd bean, or mash. It is considered as a potential crop in mitigating the nutritional problems of the developing world as it contains high protein and nutrient contents (Girish et al., 2012). However, factors such as low genetic variability, thermal sensitivity, poor harvest index, and susceptibility to biotic stresses mainly toward yellow mosaic virus, and wilt problems are some major limiting factors that constrain its yield (Kanimozhi et al., 2009).

#### Origin and taxonomy

Black gram is believed to be domesticated from its wild progenitor *V. mungo* var. *silvestris* in India (Luloki et al., 1980; Kaewwongwal et al., 2015). Indian diverse agro-ecologies and different cropping systems favor the growth of black gram under rain fed conditions. Currently, India is its largest producer and is also considered to be the primary center for black gram genetic diversity (Zeven and de Wet, 1982). However, the crop is also grown across South and Southeast Asian countries, including Thailand, Afghanistan, Philippines, Myanmar, Bangladesh, Pakistan, and Nepal (Kaewwongwal et al., 2015).

#### Domestication and global spread

Based on archeological evidences, black gram is said to be domesticated in India around 4,500 years ago in the regions of Gujarat and in the Northern Peninsula (Fuller and Harvey, 2006). *V. mungo* var. *silvestris* is considered as the wild progenitor of *V. mungo* (L.) Hepper var. *mungo* from which it would have domesticated in India (Chandel et al., 1984). It further spread from India to regions of Myanmar and Thailand (Toomaka et al., 2002; Kaewwongwal et al., 2015) (Figure 2B).

#### Genetic diversity in black gram collections

Black gram is reported to have a vast number of *ex situ* collections comprising of 6,483 accessions (http://www.fao.org/wiews). Of which, 3,153 accessions are reported to be with National Bureau of Plant Genetic Resources (NBPGR) of Indian Council of Agricultural Research (ICAR), New Delhi, India along with several other gene banks of different countries such as Thailand (1,201 accessions), Pakistan (944 accessions), Taiwan (894 accessions), Japan (449 accessions) and the United States of America (304 accessions) (Gayacharan et al., 2022). A set of 840 diverse accessions originated from different parts of India was used to dissect its genetic diversity on the basis of 29 morphological traits. Of which, many traits were qualitative in nature and governed by few genes. Therefore they represented dominant type of phenotype in the population (Gayacharan et al., 2022). Further hierarchical clustering performed using these traits showed that most of the accessions were clustered in one group despite their site of origin. This might be due to the selection of quantitative traits that is influenced by human selection as well as agro-ecological conditions. Molecular diversity has also been evaluated using markers such as inter simple sequence repeat (ISSR), RAPD (Souframanien and Gopalakrishna, 2004; Srivastava et al., 2013), AFLPs (Sivaprakash et al., 2004) and SSRs (Gupta and Gopalakrishna, 2009; Suvan et al., 2020). These studies showed that different maker systems were able to dissect distinct genetic variability within the accessions. Further, excluding duplicates helped in development of core collection of elite black gram. Kaewwongwal et al. (2015) performed a large-scale molecular diversity analysis where 534 black gram accessions collected from different geographical origins were analyzed using 22 SSR markers revealing a total of 199 alleles. The study reported that the cultivated black gram of South Asia was genetically different from the West Asian accessions. In gene diversity study, higher gene diversity was observed in wild black gram than the cultivated counterpart. Furthermore, 78.67% of the wild gene diversity was found in cultivated accessions which indicated that the domestication bottleneck effect in this legume is relatively less. They also analyzed the gene diversity in black gram using SSRs that was found to be closely related to *Vigna* species such as azuki bean, mung bean, and rice bean. Results indicated that black gram is more similar to mung bean and rice bean than the adzuki bean (Kaewwongwal et al., 2015).

#### Genetics and genomics of domestication

Genetic dissection of DRTs requires high density linkage maps developed from the mapping populations obtained by crossing wild and cultivated genotypes. Limited numbers of such genetic linkage maps have been reported for black gram (Chaitieng et al., 2006; Gupta et al., 2008). First linkage map was constructed using BC_1_F_1_ population (180 individuals) developed from crossing mutant line of large seeded accession P1 JP219132 with TC2210, a wild accession from India. A total of 61 SSR primers, 56 RFLP probe-based, 27 AFLP, and a morphological marker were used in the development of 11 LGs. The map covered a total map distance of 783 cM of the genome with 148 marker loci on 11 LGs (Chaitieng et al., 2006). Comparative analysis performed in this study revealed that linkage order of adzuki bean (Han et al., 2005) and black gram is conserved particularly on LGs 1, 2 and 5. One of the regions on LG 10 of black gram was found to be highly related to LG 1 of adzuki bean (Chaitieng et al., 2006). Gupta et al. (2008) also constructed a linkage map using F_9_ mapping population derived from a cross between TU94-2 (cultivar) and *V. mungo* var. *silvestris* (wild). The linkage map covered a total map distance of 865.1 cM with 428 markers, spread across 11 LGs. Obtained linkage map was compared with that of Han et al. (2005) and Chaitieng et al. (2006), which showed a high level of colinearity within the three maps. However, several reversal of marker order was observed between the marker pair of the Gupta et al. (2008) and Chaitieng et al. (2006) that indicated internal inversion during evolutionary divergence of black gram and azuki bean. Raizada and Souframanien (2019) dissected black gram genome for DRTs using high-quality transcriptome data generated using Illumina MiSeq from the immature seeds of wild black gram (*V. mungo* var. *silvestris*). Markers developed in this study were used to screen the wild and cultivated genotypes, which revealed significant molecular variability within wild and cultivated genotypes. Wild species showed large genetic diversity that corresponds to useful domestication-related genes.

Transcriptomic analysis involving cultivated and wild accessions can be utilized to identify gene(s) or genomic regions related to domestication which gets modified in the domestication bottleneck (Raizada and Souframanien, 2019). Somta et al. (2020) reported mapping of agronomic as well as DRTs in a population derived from a cross between mutant black gram (MOG-1) and wild black gram (*V. mungo* var. *silvestris*)*.* The MOG mutant displayed gigantic morphological characters than the wild black gram, therefore called as super domestication traits. This is due to strong effect of MOG gene that masks the effect of DRTs. Therefore, the effect of DRTs cannot be well studied using the same population.

### *Vigna radiata* (L.) Wilczek

Mung bean [*V. radiata* (L.) Wilczek] is a diploid crop (2*n* = 2× = 22) rich in nutritional protein, carbohydrate, iron, folate, vitamin C, thiamin, zinc, potassium, magnesium, copper, manganese, and phosphorus (Mubarak, 2005; Keatinge et al., 2011; Taunk et al., 2012; Dahiya et al., 2013). It is cultivated mainly in Asian countries including India, Myanmar, Indonesia, and China, accounting for 90% of its total world production (Lambrides and Godwin, 2007).

#### Origin and taxonomy

Mung bean, which is predominantly grown in Asian countries, has its wild relative found in parts of Africa, Asia, Papua New Guinea, and Australia. Taxonomical description presented in Tomooka et al. (2002) is followed as standard system to categorize mung bean within *Vigna* species. It describes subgenus *Ceratotropis* (Genus *Vigna*) consisting of 21 species including mung bean. Closest relative of mung bean, that is, *Vigna radiata* var. *sublobata* (Roxb.) Verde (syn. *Phaseolus sublobatus* Roxb.) is considered as the progenitor of mung bean, which is found in the Sub-Himalayan tracts and Western Ghats of India (Paroda and Thomas, 1987).

#### Domestication and global spread

**FIGURE 3:**
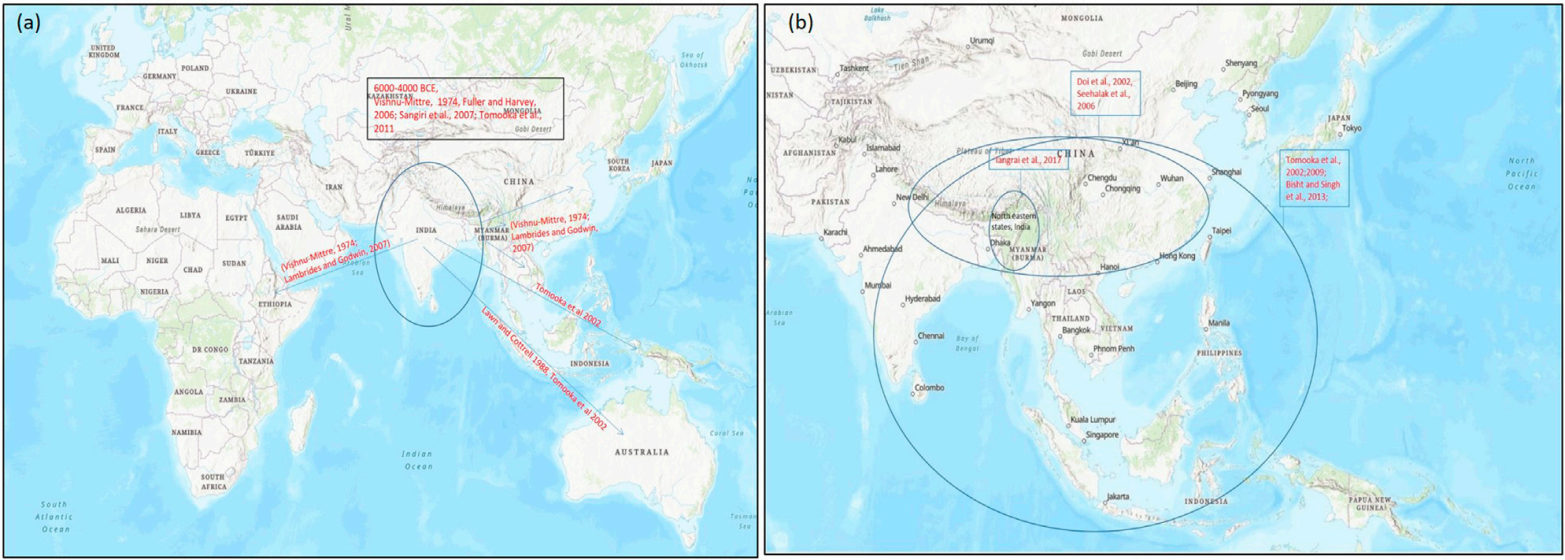
Map representing the origin of domestication of **(A)** mung bean (*Vigna radiata*) and **(B)** rice bean (*Vigna umbellata*) (encircled) and their spread (arrow) to different parts of the world on the basis of archeological evidences and genetic studies for domestication-related traits. Map source: https://www.arcgis.com/index.html.

On the basis of archeological evidences and linkage map results of wild and cultivated mung beans, it was found that mung bean has a high degree of domestication than other Asian *Vigna* species. Cultivated mung bean was domesticated in South Asia approx. 4,000–6,000 years ago from its wild crop *V. radiata* var. *sublobaba* (Roxb.) and finally adopted as a landrace in Asia under diverse environmental conditions (Fuller and Harvey, 2006; Sangiri et al., 2007; Tomooka et al., 2014) (Figure 3A). Based on archeobotany, its two domestication regions were suggested, of which one allied with the Southern Neolithic and the other with the upper Ganges basin (Fuller and Harvey, 2006). Diversity study performed by Sangiri et al. (2007) on large number of diverse mung bean genotypes supported South Asia as the major place where this crop was domesticated (Sangiri et al., 2007). Further studies on domestication and selection process showed that modern mung bean was dispersed throughout Southern and Eastern Asia, Africa, and Austronesia (Lambrides and Godwin, 2007) (Figure 3A).

#### Genetics and genomics of domestication

Molecular markers such as RFLP, RAPD, and SSRs were used to screen mapping population derived from crossing cultivated and wild mung bean genotypes. These were utilized for the construction of linkage maps to identify the QTLs and genes related to DRTs (Fatokun et al., 1992; Menancio-Hautea et al., 1993; Menancio-Hautea, 1996; Humphry et al., 2002; Humphry et al., 2003; Chankaew et al., 2011). However, these linkage maps had certain drawbacks such as they could not cover the basic number of chromosomes in mung bean (*n* = 11). Furthermore, size of mapping population was small and the developed maps were not dense for some LGs (Isemura et al., 2012). Therefore, Isemura et al. (2012) utilized BC_1_F_1_ population having 250 individuals developed from a cross between a wild accession (JP211874) of Myanmar and a cultivar JP229096 cv. Sukhothai of Thailand. In this study, they used SSRs and Expressed Sequence Tag (EST)-SSRs developed from different legume crops (329 adzuki bean, 50 mung bean, 7 cowpea, 40 common bean, 156 cowpea and 840 soybean) for detecting polymorphism between the two parents. Thereafter, polymorphic markers were used in the construction of 11 LGs which span about 727.6 cM. Also, 105 QTLs and 38 genes were identified for DRTs (Isemura et al., 2012). Among these, *Sdwa5.1.1* was found to be associated with seed dormancy.

Seed dormancy is one of the important DRTs that can be introgressed through wild genotypes of mung bean (*V. radiata* var. *sublobata*) in cultivated mung bean (var. *radiata*) to provide resistance toward pre-harvest sprouting. Laosatit et al. (2022) fine mapped locus *Sdwa5.1.1+* using F_2_ mapping population derived from a cross between ACC41 (wild) and KPS2 (cultivated). The study revealed two linked QTLs, *Sdwa5.1.1+* and *Sdwa5.1.2+* associated with seed dormancy. The QTL *Sdwa5.1.1+* contained one gene *VrKNAT7-1*, annotated as transcription factor KNOTTED *Arabidopsis thaliana*7 (KNAT7), a class II KNOTTED1-LIKE HOMEOBOX (KNOX II). Loss of function due to *MtKNOX4* mutation in *M. truncatula* is reported to be involved in reduction of hydroxylation of seed coat fatty acid. It therefore alters the cuticle layer permeability causing physical dormancy (Chai et al., 2016).

To get more genetic evidences of domestication in mung bean, pure line of cultivated mung bean *V. radiata* var. *radiata* (VC 1973A) was sequenced by Illumina HiSeq2000 to develop the first draft genome of mung bean. Homology-based study using Ortho MCL24 detected a set of 1,121 shared orthologous loci (Kang et al., 2014). Li et al. (2018) reported that wild mung bean have indeterminate stem along with apical meristems that maintained the vegetative activity, while cultivated mung bean has determinate stems and apical meristem, which maintains the reproductive activity after flower induction. The determinate plant shows early flowering and pod maturation than indeterminate plants. *VrDet1* is a single gene that modulates the indeterminate growth in wild plants and encodes the shoot apical meristem proteins. Conversion from indeterminate to a determinate stem growth habit was achieved by two linked point mutations in two putative cis-regulatory elements. *VrDet1* was found to be orthologous to *Dt1* in wild soybean and *PvTFL1y* in wild common beans (Li et al., 2018). Different studies in mung bean showed that most DRT QTLs were confined to LGs 1, 2, 4, 6, 7, 8, and 9 (Table 1).

### *Vigna umbellata* (Thunb.)

Rice bean [*V. umbellata* (Thunb.)] is a diploid (2n = 2× = 22), warm-season, annual crop which is mostly grown under sifting cultivation. Its genome size is estimated to be approximately 400 Mb (Kaul et al., 2019). In general, the crop is consumed as pulse, vegetable, folk medicine, fermented pulse and in the form of animal fodder (Iangrai et al., 2017). It is mainly cultivated in East Asian countries such as Nepal, Bhutan, Northeast India upto Myanmar, Southern China, Northern Thailand, Laos, Vietnam, Indonesia, and East Timor (Tian et al., 2013).

#### Origin and taxonomy

The origin of rice bean has been staged in South and Southwest Asian countries (Bisht et al., 2005; Singh et al., 2014) or the regions of Indo-China (Doi et al., 2002). Its wild form, that is, *V. umbellata* var. *gracilis* is reported to be found in the regions of Himalayas, Central China and Malaysia (Seehalak et al., 2006). Phylogenetic analysis of rice bean and related species has been demonstrated through several studies using biochemical and molecular markers such as isozymes, RAPD, RFLP, AFLP, ISSR and plastid DNA (Pattanayak et al., 2019). Studies based on ITS and atpB–rbcL regions DNA sequences showed that both wild and cultivated forms of *V. umbellata* are related to Angulares species such as *V. hirtella*, *V. exilis*, *V. tenuicaulis*, *V. napalensis*, *V. minima*, *V. nakashimae*, and *V. ruikiuensis* (Doi et al., 2002; Takahashi et al., 2016).

#### Domestication and global spread

Rice bean is said to be domesticated in Asia from its wild form, that is, *V. umbellata* var. *gracilis* which is a cross fertile type crop (Iangrai et al., 2017) widely distributed in natural and distressed habitat, and the tropical rain forest areas from the eastern India, Nepal, Myanmar, Thailand, Laos and Southern China to East Timor (Tomooka et al., 2002; Isemura et al., 2010; Iangrai et al., 2017). The wild form is characterized by traits such as indeterminate nature, twining plant types, free-branching, small seeds and sensitivity toward photoperiodism. The rice bean landraces are widely distributed in Northeastern India and seems to be similar to its wild forms (Iangrai et al., 2017a). Although, there is no consensus about the exact location of rice bean domestication, it is believed to have occurred in Southeast Asia owing to the fact that the region represents very high variability of rice bean landraces. A study that involved intra and interspecific diversity among the *Vigna* species from Thailand and adjoining regions suggested single domestication event for rice bean in Thailand and neighboring Shan state, Myanmar (Seehalak et al., 2006). Similarly, other studies have also showed high variability for traits including seed size in the germplasm collected from the Southeast Asian region. Based on these facts, it is agreed that rice bean may have domesticated in Southeast Asia and then spread to the other parts of the world.

#### Genetic diversity in global rice bean collections

Rice bean is naturally distributed in the wide area of tropical rain forest extending from South, Southeast Asia to East Asia. Efforts have been made to collect rice bean germplasm from these regions. The largest collections of rice bean (1920 accessions) is available in National Gene Bank of India, that is, ICAR-NBPGR, New Delhi (http://www.nbpgr.ernet.in:8080/PGRPortal/(S(5a0gla55sxz1oy555ojrl055))/SearchResult.aspx accessed on 14.08.2022) followed by National Institute of Agrobiological Sciences (NIAS), Japan (467 accessions; https://www.gene.affrc.go.jp/databases-plant_search_en.php accessed on 14.08.2022) and the Plant Genetic Resources unit, Nepal Agricultural Research Council (300 accessions; http://narc.gov.np/national-agriculture-genetic-resources-center-genebank/ accessed on 14.08.2022). The rice bean germplasm collection at the Indian National Gene bank represents accessions from all over India, however large parts of it comes from the Northeastern region, which is considered to be one of the probable centers of its origin/diversity.

Molecular markers based analysis of rice bean collection from its diversity rich regions have provided some insight into its probable center of diversity and domestication (Seehalak et al., 2006; Bajracharya et al., 2008; Tian et al., 2013). An AFLP marker based study of cultivated and wild rice bean accessions from Nepal, Japan, Thailand and Myanmar revealed that cultivated accessions were grouped with wild types. The weak differentiation between cultivated and wild rice bean accessions suggested frequent gene flow between them. Furthermore, on the basis of clustering pattern of the genotypes, it was suggested that domestication of rice bean may have occurred in the Northern Thailand and neighboring Shan state, Myanmar. However, a definite conclusion about its domestication could not be drawn since their study did not represent accessions from South Asia and China (Seehalak et al., 2006) (Figure 3B). Tian et al. (2013) reported the first comprehensive marker based diversity analysis of rice bean with 472 diverse accessions (388 cultivated and 84 wild) collected from 16 Asian countries which were evaluated using 13 SSR markers. The cultivated rice bean populations from Vietnam, Myanmar, Nepal, and India were found to be most diverse. Indonesian cultivated accessions showed high genetic divergence from other cultivated accessions and their genetic structure was more similar to wild accessions. This indicated towards the possibility for a separate domestication event of rice bean in Indonesia, however to support this view a large number of rice bean accessions from Indonesia should be analyzed. The accessions collected from Western region of Nepal showed different pattern and formed a new precise group. This group showed similarity with the South Asian rice bean. Even an earlier study that analyzed rice bean accessions from India and Nepal had found accessions from Western Nepal to be very distinct (Bajracharya et al., 2008). The rice bean accessions from West Nepal may possess useful genes and can be a great valuable genetic resource for improvement of rice bean in other countries (Tian et al., 2013).

The genetic diversity studies have suggested different centers of rice bean domestication and this could be attributed to lack of sufficient number of accessions from its whole of diversity rich regions and use of limited number of markers in various studies. For example, Tian et al. (2013) used only 13 SSR markers, which equates to slightly more than one marker per linkage group, and is insufficient for uncovering the whole genome level diversity pattern in collections of cultivated and wild accessions. Also, gene flow between wild and cultivated accessions may have confounded over all the interpretations. Therefore, in order to get a clear-cut final conclusion about the location of domestication of rice bean, it would be more appropriate to collect large number of cultivated and wild rice bean accessions from whole breadth of its diversity and analyze diversity pattern in these accessions using sufficiently large number of markers, preferably by SNP markers discovered using whole genome resequencing or GBS approaches.

#### Genetics and genomics of domestication

Trait mapping studies in rice bean are limited due to non-availability of genomic resources and lack of focus on its genetic improvement. Therefore, at present, we know very little about genetic basis of any trait including DRTs in this crop. Nonetheless, a few genetic mapping studies using AFLP or SSR markers from other *Vigna* species have been carried out in the last 10 years to uncover genomic regions/QTLs for DRTs and other desirable traits. First genetic study for DRT in this crop used mapping populations derived from interspecific crosses between rice bean × azuki bean and rice bean × *Vigna kashimae* and QTLs associated with seed weight were identified (Kaga et al., 2000; Somta et al., 2006). Later, it was Isemura et al. (2010) who undertook the first comprehensive genetic study to discover QTLs/genomic regions for 31 DRTs using a BC_1_F_1_ population derived from a cross between cultivated (JP217439) and a wild rice bean accession (JP210639). The QTLs governing DRTs were clustered into small genomic regions on chromosomes. Moreover, they also found some large phenotypic effects QTLs on LG 2 (seed and pod size), LG 4 (seed and pod size, and water absorption by seed) and LG 7 (pod dehiscence and stem length). Overall, their findings suggested simple genetic control for DRTs since few major QTLs accounted for large phenotypic variations of many DRTs between cultivated and wild rice beans. This was not surprising given that DRTs have been reported to be controlled by major genes in other crops as well (Burke et al., 2007). Although, we now have some understanding of genetic regulation of DRTs in rice bean, an integrated approach that combines conventional genetic mapping with advanced genomics tools such as pan-genome sequencing, GWAS, transcriptomics and metabolomics is required to localize exact position of domestication-related genes in rice bean genome. In this direction, Kaul et al. (2019) have generated a draft genome sequence of rice bean and annotated many genes for late flowering and palatability in rice bean by aligning its genome sequence against the genome sequences of 31 leguminous plant species. Transcriptome analysis performed by Verma et al. (2021) identified candidate genes which were associated with size and development of rice bean seeds. It is worthwhile to note that palatability is a major constraint in rice bean’s lower consumer preference limiting its wide scale adoption as a major pulse crop. Also, palatability is an important domestication target trait in rice bean. Manipulating the genomic regions associated with low palatability might increase its consumer demand.

### *Vigna aconitifolia* (Jacq.) Marechal

Moth bean (*V. aconitifolia*) is a diploid (2n = 2× = 22) crop, which is highly adapted to heat and drought stresses (Tomooka et al., 2002). It can withstand high temperature upto 45 °C with a low annual rainfall of 200–300 mm. Generally, this crop is found in arid and semi-arid regions of India, Pakistan, Afghanistan, Nepal, Sri Lanka, Myanmar, and in some African countries (Blink and Jansen, 2006).

#### Origin and taxonomy

Moth bean is a member of genus *Vigna*, subgenus *Ceratotropis* and section *Aconitifoliae*, Tomooka and Maxted (Tateishi, 1985; Bisht et al., 2005; Yadav et al., 2015; Yadav et al., 2015). Besides moth bean, five other *Vigna* species are also classified under section *Aconitifoliae*, *viz., V. aridicola* N. Tomooka & Maxted; *V. indica* T.M. Dixit, K.V. Bhat, and S.R. Yadav; *V. khandalensis* (Santapau) Raghvan et Wadhwa; *V. trilobata* (L.) Verdc.; and *V. stipulacea* (Lam.) Kuntze. Moth bean is believed to be the native of India, and this theory was also supported by various studies that have documented distribution of wild moth bean form in Southeastern India (Tomooka et al., 2009; Takahashi et al., 2016).

#### Domestication and global spread

Until recently, there was no clear-cut understanding on the wild progenitor as well as the site of moth bean domestication. In fact, initially, *Vigna trilobata* (L.) Verdc. was considered as its wild form (Whyte et al., 1953). Later, however, morphological and molecular analyses allowed researchers to clearly distinguish between wild and domesticated moth beans. Thereby, *V. trilobata* was found to be an entirely distant species (Doi et al., 2002; Saini and Jawali, 2009; Javadi et al., 2011; Takahashi et al., 2016). Molecular analysis performed on the moth bean accessions collected from Tamil Nadu, India, revealed a few accessions that were largely similar to cultivated moth bean but differed in some features and can be considered as wild progenitor of moth bean (Tomooka et al., 2009; Takahashi et al., 2016). For example, both domesticated and wild accessions have similar variations in leaflet shape but the seeds of wild accessions were covered with a semi transparent seed coat, whereas that of domesticated accessions has larger seed size with water permeable seed coat along with non-shattering pods (Takahashi et al., 2016). Furthermore, the occurrence of wild form of moth bean accessions in Southern part of India suggests that most likely the this plant would have been domesticated in this region before spreading to other regions of India and other countries.

#### Genetic diversity of moth bean collections

Moth bean cultivation area mainly falls in the arid and semi-arid regions of India, however to some extent it is also grown in Pakistan, China, Bangladesh and Myanmar. ICAR-NBPGR, New Delhi, maintains its largest collection (1,511 accessions) mainly collected form the Indian states such as Rajasthan, Gujarat, Punjab, Maharashtra, along with union territory Jammu and Kashmir (Bisht et al., 2005). Besides, smaller collections are also maintained at the USDA Southern Regional Plant Introduction Station, Griffin, Georgia, (56 accessions) and in the National Genebank of Kenya (47 accessions). However, so far, there is no systematic genetic diversity study on a large set of *V. acontifolia* germplasm collection, which is necessary for having comprehensive understanding of its origin, center of diversity and domestication. Recently, few studies have reported genetic diversity studies on small number of moth bean germplasm either using agro-morphological traits (Kohakade et al., 2020) or by using molecular markers such as ISSR (Bhadkaria et al., 2020). Molecular analysis of accessions collected from different parts of India revealed that accessions form the Northwestern regions of India are more diverse as compared to those belonging to the Western coastal province (Gujarat) of India. This suggests that Northwestern region of India may be representing the center of diversity of the domesticated moth bean (Bhadkaria et al., 2020).

#### Genomics of domestication

Moth bean is probably the most primal crop of genus *Vigna*, however, its genetics of the domestication process is not yet completely understood due to lack of genomic resources such as markers which are required for the genetic mapping in any crop. Moreover, because moth bean is grown in a limited area, most of which falls mainly in the two countries, that is, India and Pakistan, it has got little attention by researchers for genomic and trait mapping studies. Nonetheless, a recent study has reported mapping of DRTs in this crop using a total of 1,644 SSRs markers belonging to various *Vigna* species including adzuki bean, mung bean, yardlong bean and common bean (Yundaeng et al., 2019). So far, this is just one report of genetic mapping of DRTs in moth bean. In this study, a F_2_ population of 188 plants which were developed from a cross between TN67 (wild type from India) and susceptible accession ‘IPCMO056’ (cultivated type from India) was used to identify a total of 50 QTLs and 3 genes for 20 DRTs. The number of QTLs per DRT ranged from one to eight and most of these were mapped on LGs 1, 2, 4, 7 and 10. Many LGs were shown to be involved in the genetic control of important DRTs, such as seed dormancy, sheet shattering and seed size. Seven major QTLs (PVE = 20%) for these traits were found to be located on LGs 1, 4 and 7. For organ size (seed size, pod size, leaf size, and stem thickness), the QTLs were found on LGs 1, 2, 3, 4, 7, 8, 9 and 10 (Yundaeng et al., 2019).

Comparative genomic analysis of QTLs related to DRTs of moth bean revealed that some QTLs correspond to previously identified QTLs for respective traits in other *Vigna* crops (Isemura et al., 2007; Isemura et al., 2010; Isemura et al., 2012; Kongjaimun et al., 2012a). For example, there are two QTLs of pod dehiscence identified in moth bean; one each on LG 1 and LG 7. Of these, the one on LG 1 seems to correspond to those of mung bean and yardlong bean, whereas the QTL on LG 7 appears to the one identified in other *Vigna* crops. Nonetheless, there are also some large effects QTLs for DRTs that are known to be limited to moth bean only, which shows involvement of additional genes/genomic regions. This is expected considering that moth bean is adapted to arid climatic regions where other *Vigna* species cannot grow efficaciously (Yundaeng et al., 2019). Among the various DRTs in moth bean, the one trait that requires attention of the researchers is seed size, because it is smallest among all the *Vigna* species. The small seed size of moth bean may be attributed to the fact that mutation which may have contributed to large effect on seed size in other *Vigna* species was not exploited in the domestication process of moth bean.

### *Vigna vexillata* (L.) A. Rich

Zombi pea [*Vigna vexillata* (L.) A. Rich] is amongst the underutilized leguminous crop which is a rich source of quality protein and minerals and is closely related to cowpea (Ogundiwin et al., 2005). It is well adapted legume toward abiotic stresses such as drought, water logging and can also be grown under alkaline, saline and acidic soil conditions (Dachapak et al., 2017). Furthermore, it is resistant toward different economically dangerous cowpea insects and pests (Ogundiwin et al., 2005).

#### Origin and taxonomy

*Vigna vexillata* (L.) A. Rich. is categorized into subgenus *Plectotropis*, genus *Vigna* within the subtribe *Phaseolinae*. *Plectotropis* subgenus is positioned as intermediate between two subgenera, that is, *Vigna* and *Ceratotropis*. It is further grouped into two sections *Plectotropis* and *Pseudoliebrechtsia* that consist of seven species (Pienaar & Kok, 1991). *V. vexillata* shows morphological variation in terms of shape and size of leaflets, pods, and seeds and is, therefore, divided into eight taxonomic varieties namely *macrosperma*, *vexillata*, *angustifolia*, *dolichomena*, *yunnanensis*, *plurifora*, *lobatifloria*, and *ovata.* First taxonomic variety is the cultivated type, while seven others are categorized as wild type (Marechal et al., 1978; Maxted et al., 2004).

#### Domestication and global spread

*V. vexillata* is cultivated across African, Asian, American and Australian continents for its two forms, that is, seed and tuber. The cultivated seed form is said to be domesticated in tropical parts of Africa (Dachapak et al., 2017); while the domestication of tuber form is confined to parts of Southeast Asian islands such as Bali and Timor (Karuniawan et al., 2006) together with northeastern region of India (Asati and Yadav 2004).

#### Genetic diversity in zombie pea collections

Being an underutilized crop, genetic diversity of zombi pea is confined to only few studies. Molecular markers such as RAPD (Spinosa et al., 1998) and SSRs (Dachapak et al., 2017) have been used to deduce the molecular variability within this species. Spinosa et al. (1998) used 47 genotypes of different botanical origins and geographical regions to assess genetic variation with the help of 20 RAPD markers. The study clustered most of the accessions from Africa and Latin America into two distinct dendrogram clusters. Dachapak et al. (2017) genetically dissected 422 zombi pea genotypes that included 408 wild and 14 cultivated accessions using 20 SSRs. These accessions belonged to the regions of Africa, America, Australia and Asia. Overall analysis separated these accessions into three major clusters. First included accessions from America, second from Africa and Asia, and third from Australia. Furthermore, dendrogram results suggested that American and Australian accessions were originated from East African region, while African and Asian accessions were genetically distinct. Therefore, it was concluded that Africa is the center of diversity and origin of zombi pea.

#### Genetics and genomics of domestication

Limited work has been reported on the DRT-associated QTLs in zombie pea. Dachapak et al. (2018) evaluated genetics of 22 DRTs using F_2_ mapping population derived from a cross between cultivated tuber form of *V*. *vexillata* (JP235863) and wild *V*. *vexillata* (AusTRCF66514). Several genomic rearrangements were noticed in *V*. *vexillata* when compared with LGs of other four *Vigna* species *viz.* cowpea, azuki bean, mung bean, and rice bean. Major QTLs for pod length, leaf size, seed thickness and other seed-related traits were confined to LG 4, LG 5 and LG 7, respectively. Overall, conserved QTLs were detected when compared to that found in cowpea, azuki bean, mung bean and rice bean. Conserved QTLs associated with traits like seed size, pod size and leaf size were found between zombi pea and cowpea. Furthermore, QTLs associated with seed size in each species were clustered on the same LGs. Amukul et al. (2020) evaluated 13 DRTs using F_2_ mapping population derived by crossing cultivated (var. *macrosperma*) and wild (var. *vexillata*) zombi pea accessions. The result showed that LG 5 harbored major QTLs related to days-to-flowering, stem length, number of branches, pod length, 100-seed weight, seed length, and seeds per pod, while QTLs associated with seed dormancy and pod shattering were found to be linked to LG 3 and LG 11.

#### Genomic synteny and common DRT QTLs within *Vigna* species

**FIGURE 4:**
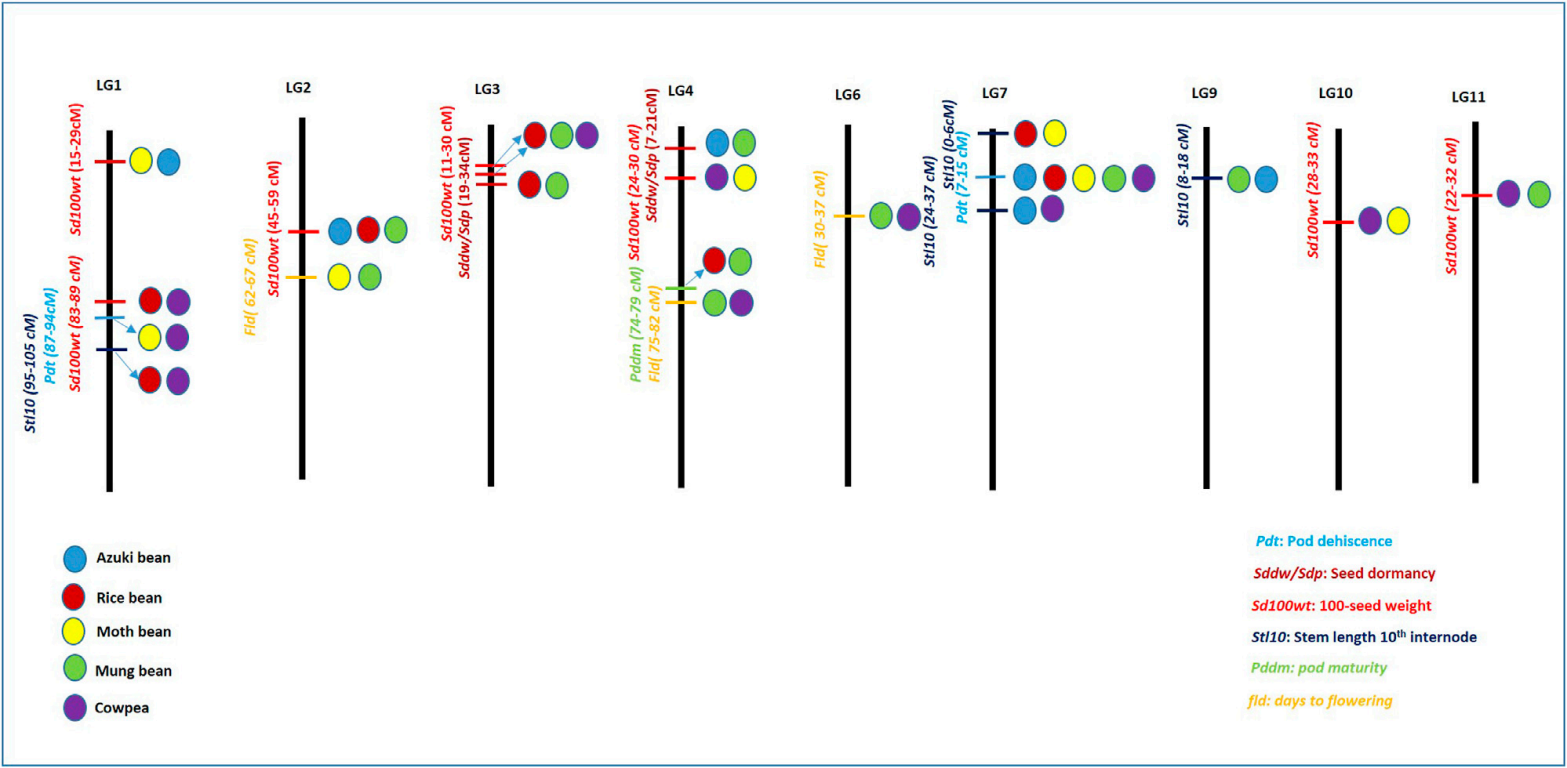
Pictographical representation of highly associated linkage groups along with common quantitative trait loci (QTLs) associated with domestication-related traits (DRTs) in *Vigna*. The length of linkage groups does not represent the scale. Differently colored circles differentiate different *Vigna* species. Blue: azuki bean, red: rice bean, yellow: moth bean, green: mung bean, and violet: cowpea.

Synteny mapping along with identification of common QTLs can reveal genomic conservation in different *Vigna* species. *Vigna* crops including azuki bean, rice bean, mung bean, black gram, and yardlong bean have been reported to have high degree of conserved genomic regions as reported by Yundaeng et al. (2019). The study reported genomic synteny within six *Vigna* species with many common markers mapped to the same LGs and in similar orders. Markers inversions were reported on LGs 1, 2, 6, 7, 8 and 11. Marker CEDG014 showed translocation when LG 1 of moth bean was compared with LG 5 of azuki bean and rice bean. Overall, moth bean, mung bean, azuki bean, and rice bean showed high degree of genomic conservation although they had different morphology, origin of domestication and environmental adaptation. In this review, we made an attempt to compare different *Vigna* species for DRTs based common QTLs. On the basis of linkage map positions having highest PVE%, major and minor QTLs related to thirteen different DRTs on different LGs were compared within five *Vigna* species, that is, azuki bean (Isemura et al., 2007), cowpea (Kongjaimun et al., 2012a), mung bean (Isemura et al., 2012), rice bean (Isemura et al., 2010) and moth bean (Yundaeng et al., 2019). These high density linkage maps included SSR and EST-SSRs associated with different *Vigna* species. Furthermore, a wide range of DRTs were also evaluated from these studies which utilized F_2_, F_2:3_ and backcross populations derived from wild and cultivated *Vigna* to deduce associated QTLs. To obtain common QTLs for any specific DRT, a difference of marker distance upto 15 cm between the two QTL positions in different *Vigna* species were assumed to be corresponding with each other. A summary of LGs and the positions of QTLs associated with 13 DRT are summarized in Table 1. Amongst these, a comparative picture of LGs harboring QTLs of six major DRTs, that is, pod dehiscence (*pd*t), seed dormancy (*sddw/sdp*), 100 seed weight (*Sd100wt*), stem length 10th internode (*Stl10*), pod maturity (*Pddm*) and days-to-flowering (*Fld*) is presented in Figure 4. QTLs associated with pod dehiscence in *Vigna* were found on LG 1 and LG 7. Similar QTLs for pod dehiscence were found on LG 1 for moth bean and cowpea species within a range of 87–94 cm. LG 7 harbored similar pod dehiscence QTLs of all the five *Vigna* species within the range of 7–15 cm. QTLs associated with seed dormancy were observed to be scattered on different LGs of *Vigna* species showing least association. However, if 15 cm position difference is considered, seed dormancy QTLs of rice bean and mung bean were found on LG 3 with positions ranging between 19–34 cm, whereas LG 4 was observed to harbor QTL of azuki bean and mung bean that corresponded to each other within a range of 7–21 cm.

QTLs of another DRT, that is, 100 seed weight was found on LGs 1, 2, 3, 4, 10 and 11 amongst different *Vigna* species (Figure 4). Two sets of similar QTLs related to seed weight were found for moth bean and azuki bean (15–29 cm) on LG 1; and QTLs of rice bean and cowpea (83–89 cm) were also found on LG 1. Similarly, LG 3 also harbored two sets of corresponding QTLs for 100 seed weight of rice bean and mung bean (23–30 cm), as well as that of rice bean and cowpea (11.5–24 cm). Trait 100 seed weight also showed strong association with LG 2, where it contained corresponding QTLs of azuki bean, rice bean and mung bean within the range of 45–59 cm. Furthermore, 100 seed weight QTLs for moth bean and cowpea were found to be associated with LG 4 and LG 10 within a range of 24.3–30 cm and 28–33 cm, respectively. Also, LG 11 harbored corresponding QTLs of mung bean and cowpea ranging between 22.6 and 40 cm (Figure 4).

QTLs associated with stem length −10th internode (stl10i) were associated with LG 1, LG 7 and LG 9. LG 1 contained corresponding QTLs of rice bean and cowpea that ranged between 95–105 cm. LG 7 contained two similar QTLs of moth bean and rice bean (0–6 cm) as well as of azuki bean and cowpea (24.4–36.9). Similar QTL of azuki bean and mung bean on LG 9 ranged between 8–18 cm. Days-to-flowering QTLs were associated with LG 2, LG 4 and LG 6. LG 2 harbored similar QTLs of moth bean and mung bean (62–67 cm). Two sets of similar QTLs of mung bean and cowpea were found on LG 4 and LG 6 that ranged between 75–82 and 30–37 cm (Figure 4). Apart from these, details of similar QTLs related to other DRTs in different *Vigna* species has been listed in Table 1. Among these, highest number of similar QTLs associated with different LGs among different *Vigna* species were found for two DRTs *viz.,* 100 seed weight and seed length (*sdl*). Higher number of QTLs for seed weight and seed size corresponds to variation in seed size of different *Vigna* species. Similar attempt to correspond important QTLs for DRTs in moth bean with other *Vigna* group is reported by Yundaeng et al. (2019). Present section of review can help in selection of QTLs for introgression of desired QTLs from one *Vigna* species to other for crop improvement and genome reconstruction of Asian *Vigna* species.

### Orthologous genes in *Vigna* domestication

Domestication genes have been functionally preserved through ancient times and have matching, though not identical sequences in many species. Therefore, in the recently evolved *Vigna* species, domestication is an ideal mode for understanding evolutionary course of action. Pootakham et al. (2021) assessed chromosome evolution within the Phaseoloid clade and found that 7 *V. mungo* chromosomes displayed a one-to-one association with *V. radiata* and *V. unguiculata* genomes. Six *V. mungo* chromosomes showed a one-to-one association with *V. angularis* and *Phaseolus vulgaris* (common bean) genomes. Furthermore, 2 *V. mungo* chromosomes demonstrated one-to-two syntenic association with *Glycine max* (soybean) due to the whole genome duplication event in the *Glycine* genome. Similarly, in a study by Lonardi et al. (2019), *V. unguiculata* chromosomes Vu02, Vu03, and Vu08 revealed association with *V. angularis* and *V. radiata* and one-to-two association with *P. vulgaris*. These studies advocate that these chromosomal reorganizations are representative of the deviation of *Vigna* from *Phaseolus*.

Agrarian traits caught throughout domestication are often coded by few loci with major phenotypic effects. It is usual to catch that these loci have putative orthologous equivalents in other species. Rajappa et al. (2016) have categorized a collection of 230 conserved ortholog set (COS) markers utilizing ESTs from four *Vigna* species, and it was observed that several among those amplified in *V. radiata* can function as anchor markers for the syntenic maps of other *Vigna* species. During deducing the draft genome sequence of *V. angularis*, Kang et al. (2015) judged orthologs among cultivated and wild adzuki bean to expound domestication-related loci. Furthermore, it was found that *V. angularis* var. *angularis* is tightly interrelated to *G. max* exhibiting collinearity of maximum genes. Furthermore, they interpreted the genomic locations of 2010 QTL-linked SSRs of *G. max* to parallel genomic locations of *V. angularis* by 569 orthologous synteny blocks. Similarly, in *V. umbellata* draft genome sequence, late flowering and unpalatability related genomic reserves for effectual domestication have been deciphered (Kaul et al., 2019). In this study, out of 31,276 total mapped rice bean genes, orthologous loci were found by way of gene mapping for 16,892 genes to *V. angularis*, 19,640 loci for *V. radiata* and 17,989 loci for *V. unguiculata*.

Functional maintenance of orthologous genes in domestication and enhancement has been extensively uncovered (Ye and Fan, 2021). Vittori et al. (2021) classified orthologous genes in *P. vulgaris* that in other species have essential functions in alteration of pod shattering, cell wall reformations, and putative pod-shattering-associated functions. In this study, BC_4_/F_4_ introgression lines derived from a cross between a domesticated genotype Midas (indehiscent) and a wild genotype namely G12873 (highly shattering) was used to narrow down the major QTL related to pod indehiscence, that is, qPD5.1-*Pv*. Differential expression analysis of specific genes confined to this locus was also performed for candidate gene identification. Of which, gene *Phvul.005G157600* located 11 kb downstream to significant peak was identified orthologous to *AtMYB26*. The function of *AtMYB26* related to anther dehiscence and secondary cell wall differentiation has also been established in *A. thaliana* (Yang et al., 2007)*.* In a recent report, *MYB26* gene has been found to be associated with pod shattering in mung bean. Two copies of this gene, that is, *VrMYB26a* and *VrMYB26b* were reported to be located on chromosome five and nine. Lower expression of *VrMYB26a* in mung bean cultivars reported in this study reduced the expression of gene associated lignin biosynthesis. Hence, cultivar showed non-twisting pods with thinner sclerenchyma layer that reduced pod shattering (Lin et al., 2022). Furthermore, Takahashi et al. (2019) testified that pod shattering as well as tenderness is related with MYB26 orthologues in *V. angularis* and *V. unguiculata*. Another important DRT *viz.* stem growth habit is a vital plant architectural feature defining yield capacity in pulses. Also, phenotypic transformation from the indeterminate stem growth habit of wild mung bean to the determinate habit of cultivated ones is a significant domestication switch. Indeterminate habit in wild is controlled by a single gene, *VrDet1*, which encrypts a signaling protein of shoot apical meristems. This gene was orthologous to *Dt1* in wild soybean and *PvTFL1y* in wild common bean (Li et al., 2018).

During genomic delineation of DRTs in *V. angularis*, a QTL for seed weight was noticed on LG 1 at a position parallel to that of a QTL for this trait on LG 2 in *V. unguiculata* and *V. radiata*. QTLs for this trait were also perceived at similar sites on *V. angularis* LG 9 and *V. radiata* LG I (Isemura et al., 2007). In a study by Kongjaimun et al. (2012b), QTL for this trait in *V. unguiculata* was found to be located on LG 1 at a position complementary to that of a QTL on LG 2 in *V. unguiculata* and *V. radiata*. Other QTLs for seed weight were also found at similar locations on LG 4 of *V. unguiculata* ssp. *sesquipedalis* (yardlong bean) and LG 6 of *V. unguiculata*, as well as LG 8 and LG 10 of yardlong bean and LG 3 and LG 4 of mung bean. In a study by Akito et al. (2011), QTLs for numerous DRTs of *V. angularis* were found to be bundled on LGs 1, 4, 7 and 9. Similar bunches were located on *V. umbellata* LGs 1 and 7. Also, orthologous QTL with a huge impact for loss of seed shattering for *V. angularis* and *V. umbellata* was discovered on LG 7. Seed shattering in case of *V. radiata* is regulated partly by the same QTL with minor effect and additional QTL on LG 1. On the contrary, *V. mungo* possessed a dissimilar QTL with minor effect on LG 5. Furthermore, an orthologous QTL governing flowering time between *V. radiata* and *V. angularis* was found on LG 4.

When linkage map of *V*. *vexillata* (zombi pea) developed by Dachapak et al. (2018) was matched with the previous linkage maps of *Vigna* species, it was revealed that LGs and sequence of the markers within the *Vigna* species were quite similar. Majority of the QTLs for seed-related attributes in zombie pea were positioned onto LG 7, where several such QTLs were detected for *V. unguiculata* ssp. *sesquipedalis*, *V. angularis*, *V. radiata* and *V. umbellata*. Three QTLs, *Pddm2*.*1−*, *Sdt8*.*1+*, and *Sdnppd9*.*1−* were positioned to parallel regions in other *Vigna* species. Amkul et al. (2019) have evidenced that *V. vexillata* genome and the genomes of *P. vulgaris* and *G. max* are extremely preserved. Amkul et al. (2020) have detected *Vigun07g094201* and *Vigun07g096300* in *V. vexillata* as the candidate genes for qSdnppd7.1, which is a yield DRT associated QTL. *Vigun07g094201* sequence was found to be highly similar to *Arabidopsis* *REM39*.

### Challenges and future prospects

*Vigna* species faces major challenges from biotic and abiotic stresses that limit their overall production. Domesticating new varieties resilient toward these stresses along with yield stability is the need of the hour. Substantial phenotypic variability between wilds and its decedents can be exploited for adaptation to a new or specific environment(s). In-depth dissection of domestication process in *Vigna* is in its preliminary stage and is confined to some prominent species only such as adzuki bean, cowpea and mung bean. Selection of crops including *Vigna* species for early domestication process was relied upon identification of DRTs such as plant height, seed size, etc. These traits were mostly visual identification of plant and dependent upon external environmental conditions. Therefore, these DRTs cannot be solely relied upon to identify stable traits for domestication. Profound dissection of *Vigna* genome is essential to identify specific gene/regions that govern DRTs. To overcome these limitations, modern era of domestication has now shifted toward genetic and molecular aspects of DRTs. Genes related to domestication syndrome in *Vigna* has been identified through population genomic approaches such as biparental QTL mapping and association studies. Several domestication-related QTLs in *Vigna* are associated with SNP/SSRs markers. These markers associated with domestication syndrome can be exploited in marker-assisted selection and breeding for crop improvement. With the advent of NGS and its advancements, whole genome trancriptomics analyses involving wild accessions and their decedents can be accomplished to identify SNPs and candidate genes related to DRTs as performed in case of maize and tomato (Hufford et al., 2012; Koenig et al., 2013). Recently, genome editing technologies have gained pace and are presently used in functional genomics. Clustered regularly interspaced short palindromic repeat (CRISPR)/CRISPR associated protein 9 (Cas9) can be used to target specific genomic regions/genes associated with DRTs to make knockouts and study the functional aspects of specific gene(s). Several targeted genes of wheat (Zhang et al., 2016), cucumber (Hu et al., 2017), tomato (Zsögön et al., 2018), and rice (Zeng et al., 2020) have been altered to enhance specific DRTs. Tomooka et al. (2014) has discussed the possibilities of domesticating wild accessions of edible *Vigna* to abiotic stress adapted cultivars through ‘neo-domestication’. This process involves the mutation of targeted gene(s) related to domestication in wild through conventional mutation breeding and screened through Targeting Induced Local Lesions IN Genomes (TILLING). Takahashi et al. (2019) demonstrated *de novo* domestication of wild *Vigna stipulacia* through mutation breeding to produce mutants with reduced seed dormancy and pod shattering. Resequencing of wild accessions of *Vigna* species and their decedents can be performed to identify genetic sweep and genomic regions associated with DRTs as performed in case of pigeonpea, buckwheat, soybean, and barley (Varshney et al., 2017; Pankin et al., 2018; Zhang et al., 2021). Collective integration of these technologies will pave the path for modern domestication process for crop improvement in *Vigna* species.

## Conclusion

**FIGURE 5:**
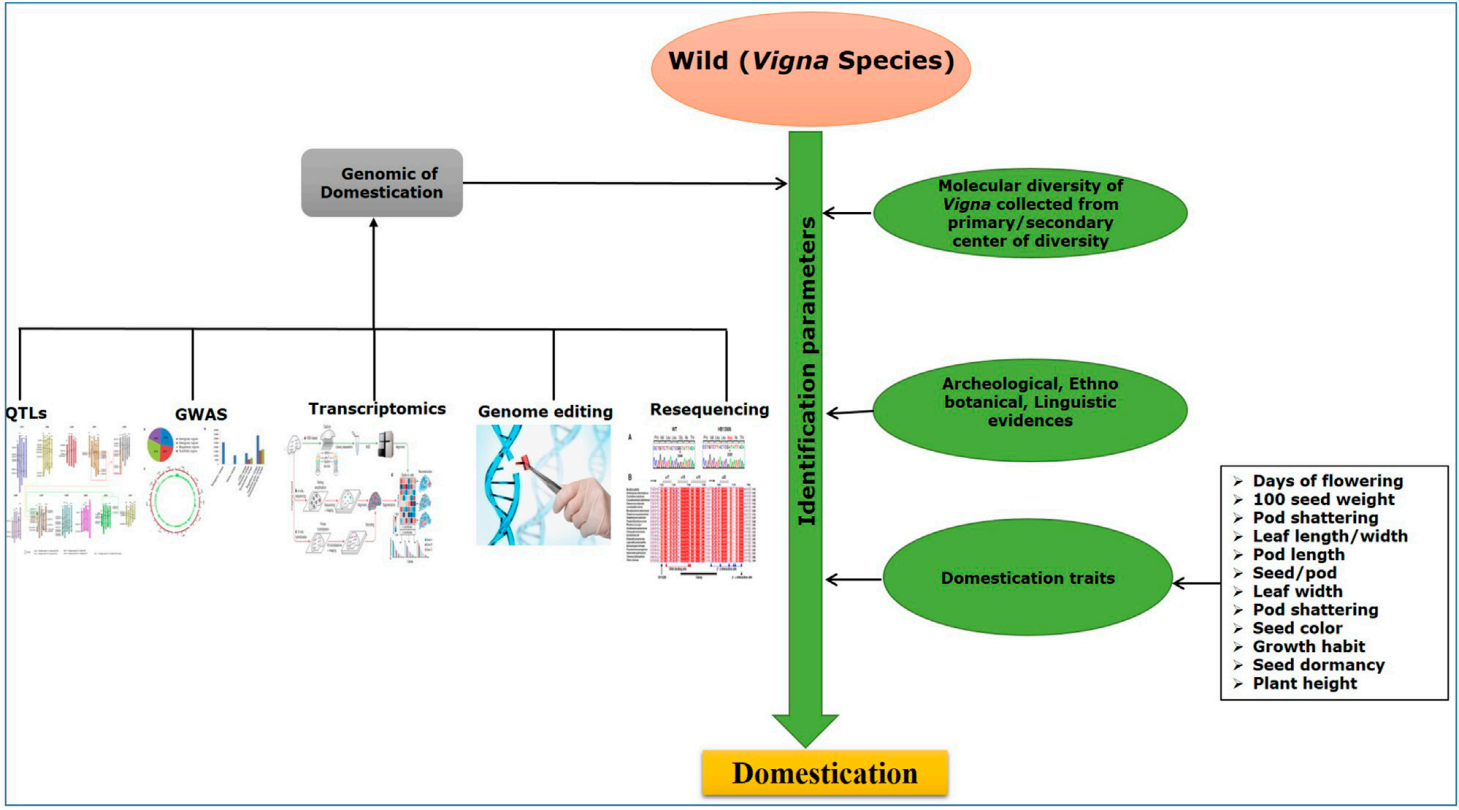
Schematic representation of various domestication-related identification parameters in *Vigna* species *viz.,* primary/secondary center of diversity; ethnobotanical and linguistic approaches; domestication-related traits (DRTs); and genomic analysis that can differentiate wilds from their domesticated counterparts.

A broad array of DRTs, site of domestication, associated genetics, genetic diversity, and genomics of seven *Vigna* species are presented in this review (Figure 5). Archeological evidences suggest that on the basis of the origin of domestication, *Vigna* species were majorly confined to warm tropical and temperate countries of East Asia, Southeast Asia, Indian subcontinent, and Northeast Africa, of which, azuki bean is evidenced to be domesticated in East Asia prominently in Japan while cowpea in Northeastern African countries such as Egypt and Sudan. Rice bean showed origin of domestication in Southeast Asian countries such as Myanmar, Thailand, and Indonesia, whereas mung bean, black gram, and moth bean showed their origin of domestication from Indian subcontinent countries predominantly in India. Traits such as seed shattering, dormancy, seed coat, alteration in seed size, seed color, maturity, and pod dehiscence that categorized wild from its domesticates were studied as DRTs in these *Vigna* species. As these traits are influenced by environmental conditions, therefore, molecular markers that can efficiently distinct genetic variability amongst different accessions were used for detection of elite genotypes. It further helped in fishing out duplicates in germplasm collection and helped in selection of core set that enhanced germplasm management for use in breeding and conservation of *Vigna* species. Genotypes having high molecular variability can be used in development of mapping population that in turn can help in detection of gene(s) and QTLs related to domestication. Presently, most of the mapping population derived from the cross between wild and cultivated genotypes to study QTLs related to domestication in *Vigna* species are of F_2_, F_2:3_, or backcross generation. Efforts can be made to map domestication-related QTLs under more stable mapping populations such RILs that can confirm the stability of mapped QTLs. Furthermore, most of the DRTs were controlled by more than one QTL that occurs on different genetic LGs. Different LGs were found to be associated with the DRTs across *Vigna* species, of which LG 1, 2, 3, 7, and 9 were the common LGs where QTLs of DRTs were identified. Several candidate genes, such as *MYB26*, *MYB83*, Knotted *Arabidopsis thaliana*7 (KNAT7), and CESA7 associated with these QTLs can be used for functional studies to reveal major factors related to DRTs. Alterations of these candidate genes through gene editing or introgression through breeding strategies can be used for development of high yielding varieties of *Vigna*. Sequencing technologies are widely used to understand the genomic complexity of *Vigna* species. As of now, *V. unguiculata*, *V. radiata*, and *V. angularis* whole genomes have been published, whereas two draft genomes of adzuki bean (draft 75% and draft 83%), one draft genome of mung bean (draft 80%), and one draft genome of rice bean (draft 96.08%) have been published. Study of DRTs using molecular markers is in its initial stage in genus *Vigna*, of which, moth bean is least studied amongst all. Thus, further genomic dissection of *Vigna* species especially moth bean will enhance our understanding about DRTs in *Vigna* species. Information provided in this review will be helpful in developing new *Vigna* varieties and in crop development programs.
